# Circulating Non-coding RNAs in Renal Cell Carcinoma—Pathogenesis and Potential Implications as Clinical Biomarkers

**DOI:** 10.3389/fcell.2020.00828

**Published:** 2020-09-15

**Authors:** Dominik A. Barth, Rares Drula, Leonie Ott, Linda Fabris, Ondrej Slaby, George A. Calin, Martin Pichler

**Affiliations:** ^1^Research Unit of Non-Coding RNAs and Genome Editing, Division of Clinical Oncology, Department of Internal Medicine, Comprehensive Cancer Center Graz, Medical University of Graz, Graz, Austria; ^2^Department of Translational Molecular Pathology, The University of Texas MD Anderson Cancer Center, Houston, TX, United States; ^3^Research Centre for Functional Genomics and Translational Medicine, Iuliu Hatieganu University of Medicine and Pharmacy, Cluj-Napoca, Romania; ^4^Department of Tumor Biology, Center of Experimental Medicine, University Medical Center Hamburg-Eppendorf, Hamburg, Germany; ^5^Central European Institute of Technology, Masaryk University, Brno, Czechia; ^6^Department of Comprehensive Cancer Care, Masaryk Memorial Cancer Institute, Brno, Czechia; ^7^Department of Experimental Therapeutics, The University of Texas MD Anderson Cancer Center, Houston, TX, United States

**Keywords:** biomarker, liquid biopsy, prognosis, long non-coding RNA, microRNA, prognosis, diagnosis, renal cell carcinoma

## Abstract

Liquid biopsy—the determination of circulating cells, proteins, DNA or RNA from biofluids through a “less invasive” approach—has emerged as a novel approach in all cancer entities. Circulating non-(protein) coding RNAs including microRNAs (miRNAs), long non-coding RNAs (lncRNAs), and YRNAs can be passively released by tissue or cell damage or actively secreted as cell-free circulating RNAs, bound to lipoproteins or carried by exosomes. In renal cell carcinoma (RCC), a growing body of evidence suggests circulating non-coding RNAs (ncRNAs) such as miRNAs, lncRNAs, and YRNAs as promising and easily accessible blood-based biomarkers for the early diagnosis of RCC as well as for the prediction of prognosis and treatment response. In addition, circulating ncRNAs could also play a role in RCC pathogenesis and progression. This review gives an overview over the current study landscape of circulating ncRNAs and their involvement in RCC pathogenesis as well as their potential utility as future biomarkers in RCC diagnosis and treatment.

## Introduction

Renal cell carcinoma (RCC) makes up for more than 90% of malignant kidney tumors, which measures up to 338,000 newly diagnosed patients each year worldwide and makes RCC the second most common urogenital malignancy in men and the third most frequent in women (Ferlay et al., 2015; Znaor et al., 2015; Siegel et al., 2019). The most frequent histological subtype is clear cell renal cell carcinoma (ccRCC) accounting for 70–80%, followed by papillary and chromophobe RCC (Rini et al., 2009). Roughly 20–30% of patients are diagnosed with an initially metastatic disease (Janzen et al., 2003), which only allows palliative treatment strategies (Powles et al., 2017). Although patient outcomes in these treatment settings have significantly improved since the introduction of immune checkpoint inhibitor therapy and combinations with antiangiogenetic drugs, most patients eventually experience a progressive disease (Motzer et al., 2015, 2018; Cella et al., 2019; Rini et al., 2019).

To date, there are no screening programs or reliable and economically reasonable biomarkers available for the early diagnosis of RCC that would drastically improve the disease outcomes (Rossi et al., 2018). Moreover, although many easily available blood-based biomarkers for the prognosis and the prediction of treatment outcome have been assessed in different stages of treatment and disease, they have not entered clinical routine yet (Dalpiaz et al., 2017; Farber et al., 2017; Seles et al., 2017). In the recent years, liquid biopsies such as circulating tumor cells (CTCs) or circulating ribonucleic acids, obtained from, for instance, blood or urine samples, are emerging (Ellinger et al., 2016; Li G. et al., 2017; Bergerot et al., 2018; Cimadamore et al., 2019). These liquid biopsies serve as novel and promising tools for the diagnosis, prognosis prediction, and selection of appropriate treatment options in RCC patients (Cimadamore et al., 2019; Smith et al., 2020).

Circulating non-coding RNAs (ncRNAs), including microRNAs (miRNAs) and long non-coding RNAs (lncRNAs), have been repeatedly suggested as well-accessible blood-based biomarkers in numerous cancer entities, including urogenital malignancies such as prostate (Su et al., 2015), bladder (Nandagopal and Sonpavde, 2016), testicular (Dieckmann et al., 2019), and kidney cancer (Ellinger et al., 2016). miRNAs are a class of non-protein coding RNAs that are about 20 nucleotides in length (Schwarzenbacher et al., 2019). They bind to the mRNA of their target proteins at the 3′UTR, which results in limited translation and increased degradation of the mRNA (Bartel, 2004). Thus, miRNAs are regulators of many cell functions including cell growth and proliferation and therefore play a principal role in tumorigenesis and progression of RCC and thus may represent useful biomarkers (Schanza et al., 2017; Barth et al., 2019). On the other hand, lncRNAs are over 200 nucleotides in length and regulate cellular processes by different mechanisms (Novikova et al., 2013). They sponge miRNAs, thereby preventing them from binding to their respective targets, which leads to the preserved expression of these targets. Furthermore, they can act as scaffold and decoy for miRNAs or proteins or can act as guides for proteins to influence gene transcription. lncRNAs were demonstrated to regulate cancer development and progression as either oncogenic or tumor-suppressive factors (Chi et al., 2019), and thus therapeutic concepts utilizing the key role of lncRNAs in cancer have emerged over the last decade (Pan et al., 2018b; Pichler et al., 2020).

In RCC, the utility of circulating ncRNAs as biomarkers has been previously evaluated (Ellinger et al., 2016); nevertheless, in the recent years, further evidence has been added to this emerging field. The present review aims to give an update and overview on the progress of circulating ncRNAs as biomarkers across various treatment settings in RCC and highlights pathogenetic implications (see Tables 1, 2).

**TABLE 1:** Circulating miRNAs as diagnostic biomarkers in renal cell carcinoma.

miRNA/panel	Tissue expression pattern	Liquid biopsy expression pattern	Sample type (liquid biopsy)	Sample size (liquid biopsy)	Validation cohort	RCC subtypes included	Diagnostic capability	References
miR-210	↑	↑	Serum	68 patients 42 controls	NA	ccRCC	AUC = 0.874, Sen 81.0%, Spe 79.4%	Zhao et al., 2013
	↑	↑	Serum exosomes	40 patients 30 controls	NA	ccRCC	AUC = 0.8779, Sen 82.5%, Spe 80.0%	Wang X. et al., 2018
	↑	↑	Serum	34 patients 23 controls	NA	ccRCC	AUC = 0.77, Sen 65%, Spe 83%	Iwamoto et al., 2014
	NA	↑	Plasma	66 patients 67 controls	NA	ccRCC	AUC = 0.6775, Sen 89.55%, Spe 48.48%	Chen et al., 2018a
	NA	↑	Serum exosomes	82 patients 80 controls	NA	ccRCC	AUC = 0.69 Sen 70%, Spe 62.2%	Zhang et al., 2018
	NA	↑	Serum	195 patients 100 controls	NA	ccRCC, pRCC, chRCC	AUC = 0.74	Fedorko et al., 2015
miR-378	NA	↑	Serum	195 patients 100 controls	NA	ccRCC, pRCC, chRCC	AUC = 0.82	Fedorko et al., 2015
	NA	↑ (not confirmed in the validation cohort)	Serum	25 patients 25 controls	117 patients 123 controls	ccRCC	NA	Hauser et al., 2012
	NA	↑	Serum	15 patients 10 controls	90 patients 30 controls	ccRCC pRCC chRCC	AUC = 0.71, Sen 70%, Spe 60%	Redova et al., 2012
miR-451	NA	↓	Serum	15 patients 10 controls	90 patients 30 controls	ccRCC pRCC chRCC	AUC = 0.77, Sen 81%, Spe 77%	Redova et al., 2012
miR-144-3p	↑	↑	Plasma	106 patients 28 angiomyolipomas 123 controls	NA	ccRCC	AUC = 0.91, Sen 87.1%, Spe 83.02%	Lou et al., 2017
miR-508-3p	NA	↓	Serum	10 patients 10 controls	85 patients 35 controls	ccRCC	AUC = 0.80	Liu et al., 2019a
miR-885-5p	NA	↑	Serum	10 patients 10 controls	85 patients 35 controls	ccRCC	AUC = 0.87	Liu et al., 2019a
miR-224	NA	↑	Plasma	66 patients 67 controls	NA	ccRCC	AUC = 0.6056, Sen 88.60%, Spe 40.91%	Chen et al., 2018a
miR-625-3p	↑	↑	Serum	50 patients 74 controls	NA	ccRCC	AUC = 0.792, Sen 70.3%, Spe 80.0%	Zhao et al., 2019a
miR-1233	NA	↑	Serum	30 patients 30 controls	84 patients 93 controls	ccRCC, pRCC, chRCC, sRCC	AUC = 0.588, Sen 77.4%, Spe 37.6%	Wulfken et al., 2011
	NA	↑	Serum exosomes	82 patients 80 controls	NA	ccRCC	AUC = 0.82 Sen 81%, Spe 76%	Zhang et al., 2018
miR-21	NA	↑	Serum	30 patients 30 controls	NA	ccRCC	AUC = 0.865, Sen 77.3%, Spe 96.4%	Tusong et al., 2017
miR-106a	NA	↑	Serum	30 patients 30 controls	NA	ccRCC	AUC = 0.819, Sen 86.7%, Spe 70%	Tusong et al., 2017
**Diagnostic miRNA panels**								
miR-210 miR-378	NA	NA	Serum	195 patients 100 controls	NA	ccRCC, pRCC, chRCC	AUC = 0.85, Sen 80%, Spe 78%	Fedorko et al., 2015
miR-378 mirR-451	NA	NA	Serum	15 patients 10 controls	90 patients 30 controls	ccRCC, pRCC, chRCC	AUC = 0.86, Sen 81%, Spe 83%	Redova et al., 2012
miR-193a-3p miR-362 miR-572 miR-28-5p miR-378	NA NA NA NA NA	↑ ↑ ↑ ↓ ↓	Serum	28 patients 28 controls	79 patients 79 controls	ccRCC	AUC = 0.807, Sen 80%, Spe 71%	Wang et al., 2015
miR-508-3p miR-885-5p	NA	↓ ↑	Serum	10 patients 10 controls	85 patients 35 controls	ccRCC	AUC = 0.90	Liu et al., 2019a
miR-224/miR-141 ratio	NA	NA	Plasma	66 patients 67 controls			AUC = 0.9898, Sen 97.06%, Spe 98.53%	Chen et al., 2018a

*RCC, renal cell carcinoma; ccRCC, clear cell renal cell carcinoma; pRCC, papillary renal cell carcinoma; chRCC, chromophobe renal cell carcinoma; sRCC, sarcomatoid renal cell carcinoma; NA, not applicable; AUC, area under the curve; Sen, sensitivity; Spe, specificity.*

**TABLE 2:** Circulating miRNAs as prognostic biomarkers in renal cell carcinoma.

miRNA/panel	Sample type (liquid biopsy)	Sample size (liquid biopsy)	Validation cohort	RCC subtypes included	Endpoint	Outcome (high expression)	Hazard ratio (HR), 95% confidence interval (95%CI), *p*-value	References
miR-221	Plasma	43 patients	NA	ccRCC, others (not specified)	OS CSS	Poor	CSS: HR = 10.7, 95%CI 1.33–85.65, *p* = 0.024	Teixeira et al., 2014
miR-224	Serum exosomes	108 patients	NA	Not specified	PFS CSS OS	Poor	PFS: HR = 11, 95% CI 3.3–68.7, *p* ≤ 0.0001 CSS: HR = 1.6, 95% CI 1.1–2.5, *p* = 0.014 OS: HR = 9.1, 95% CI 1.8–166.1, *p* = 0.0043	Fujii et al., 2017
miR-26a-1-3p	Plasma exosomes	44 patients	65 patients	Metastatic ccRCC, pRCC, chRCC, unspecified	OS	Good	HR = 0.43, 95% CI 0.1–0.84, *p* = 0.025	Du et al., 2017
miR-615-3p	Plasma exosomes	44 patients	65 patients	Metastatic ccRCC, pRCC, chRCC, unspecified	OS	Good	HR = 0.36, 95% CI 0.11–0.54, *p* = 0.0007	Du et al., 2017
miR-let-7i-5p	Plasma exosomes	44 patients	65 patients	Metastatic ccRCC, pRCC, chRCC, unspecified	OS	Good	HR = 0.49, 95% CI 0.21–0.84, *p* = 0.018	Du et al., 2017
miR-150	Plasma	94 patients	NA	ccRCC	CSS	Good	HR = 1.3, 95% CI 1.0–1.8, *p* = 0.03	Chanudet et al., 2017
miR-210 miR-221 miR-1233	Plasma	54 patients	NA	ccRCC, Others (not specified)	CSS	Poor	HR = 3.02, 95% CI 1.19–7.64, *p* = 0.014	Dias et al., 2017

*RCC, renal cell carcinoma; ccRCC, clear cell renal cell carcinoma; pRCC, papillary renal cell carcinoma; chRCC, chromophobe renal cell carcinoma; NA, not applicable; HR, hazard ratio; 95%CI, 95% confidence interval; OS, overall survival; PFS, progression-free survival; CSS, cancer-specific survival.*

### Challenges of Circulating ncRNAs as Biomarkers in Cancer

**FIGURE 1:**
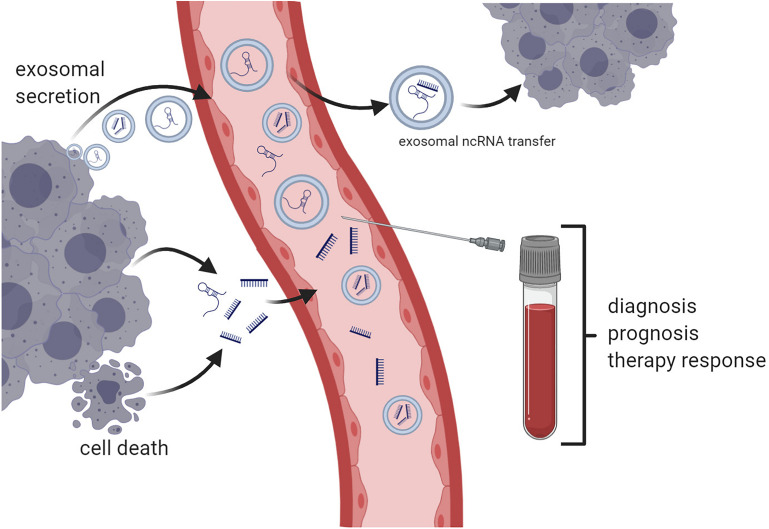
Principles of circulating non-coding RNA (ncRNA)-based liquid biopsy. Non-coding RNA products such as microRNAs and long non-coding RNAs are either actively secreted or released in the course of cell death. Exosomal ncRNAs can be transferred between different tumor sites and thereby disseminate metastatic potential (*e*.*g*., miR-19b-3p) or drug resistance (*e*.*g*., lncRNA ARSR). Blood samples may be used for diagnosis or prediction of prognosis and response to cancer therapies (created with Biorender.com).

As of yet, the development and the use of circulating RNA-based biomarkers comes with certain challenges (Zaporozhchenko et al., 2018). In contrast to cell-free DNA, which is mainly set free in the course of cell death, the largest proportion of circulating RNAs is actively secreted by normal and tumor cells and therefore could reflect current cellular processes and states to disease better, although they can be released during apoptosis and other types of cell death as well (Javidi et al., 2014; Zaporozhchenko et al., 2018). In its nature, cell-free RNA molecules are rather unstable in biofluids such as blood and urine, among others, due to the underlying environmental conditions, including pH and the enzymatic activity of ribonucleases (Tzimagiorgis et al., 2011; Bryzgunova and Laktionov, 2015; Koczera et al., 2016; Zaporozhchenko et al., 2018); nevertheless, miRNAs are astonishingly stable under these extreme conditions (Cortez et al., 2011). Interestingly, in cancer, the activity of ribonucleases in the blood may be decreased, granting a higher stability of circulating RNA products (Huang et al., 2014). Circulating RNAs are often encapsulated in microvesicles, such as exosomes or apoptotic bodies, or can be bound to lipoproteins, such as Ago2 or high-density lipoprotein, in order to protect them from degeneration (Figure 1) (Arroyo et al., 2011; Gallo et al., 2012; Wagner et al., 2013; Javidi et al., 2014; Zaporozhchenko et al., 2018).

Exosomes act as an intercellular communication channel, with signals encrypted in the form of the encapsulated DNA, RNA, or protein cargo. The composition of this cargo is highly cell specific and can be a status indicator of cellular homeostasis of the originating cell. Thus, the presence of a specific exosomal cargo signature, specifically miRNA composition, has been previously indicated as having indicative biomarker potential in a number of oncological diseases, including renal carcinoma (Butz et al., 2016; Du et al., 2017). Exosomes participate in intercellular communication and several cellular processes, including the regulation of angiogenesis, apoptosis, and metastasis, and may be utilized as biomarkers or therapeutic targets (Gurunathan et al., 2019; Zhang and Yu, 2019). Exosomes are actively secreted by most cell types through the endosomal sorting complexes required for transport pathway (Colombo et al., 2013), resulting in the fusion of the late endosomal multivesicular body and the cellular membrane (Trajkovic et al., 2008). Moreover, exosomes may transfer miRNAs and lncRNAs between tumor cells, thereby transferring drug resistance and metastatic potential as there is cumulative proof regarding the increased secretion and the differential miRNA signature identified in tumor-derived exosomes when compared to normal cells (Vojtech et al., 2014). While the miRNA sorting mechanisms have not been completely elucidated, recent insight points toward the interaction with a series of RNA binding proteins, such as members of the heterogeneous nuclear ribonucleoprotein family that have been proven to form vesicle-specific miRNA–protein complexes (Villarroya-Beltri et al., 2013). Other proposed mechanisms are based on the activity of the neutral sphingomyelinase 2 and the affinity of miRNAs to membrane raft-like structures containing ceramide as a possible loading mechanism (Groot and Lee, 2020). While it is not yet clear whether the currently proposed miRNA exosomal sorting mechanisms have any clinical relevance, the presence of specific miRNA signatures is indicated to have both mechanistic (Squadrito et al., 2014) and biomarker implications in cancer (Qu et al., 2016; Hosseini et al., 2017; Jiang N. et al., 2019; Wang et al., 2019).

As of yet, when considering cell-free circulating ncRNAs as blood-based biomarkers, differences in the study designs, including the choice of samples (serum versus plasma) and analyzing methods such as quantitative real-time PCR (qRT-PCR), next-generation sequencing, or microarrays, should be taken into account as they can heavily influence experimental outcomes (Lee et al., 2017). For instance, the total concentration of RNA extracted from samples is higher in serum as in plasma, which might be due to differences in processing, leading to the release of RNA when the blood is coagulating (Wang et al., 2012; Lee et al., 2017). Last but not least, differences in normalization of ncRNA measurements to molecules that are consistently expressed in healthy individuals and cancer patients can influence experimental outcomes. In addition, differences in sample collection, storage, and processing may impact the outcome of studies which, once again, impedes the comparability between studies, underlining the necessity of validation studies (Lee et al., 2017).

## Circulating miRNAs in RCC

### Circulating miRNAs as Potential Diagnostic Tools

Zhao et al. (2013) first discovered the potential of miR-210 in the diagnosis of RCC. Besides the upregulation of miR-210 in malignant tissues as compared to healthy adjacent parenchyma, they also discovered significantly increased miR-210 levels in serum samples of RCC patients as compared to healthy controls (Zhao et al., 2013). These results were later confirmed by several studies which found increased serum and plasma miR-210 levels in RCC (Iwamoto et al., 2014; Fedorko et al., 2015; Liu T. Y. et al., 2016; Dias et al., 2017; Chen et al., 2018a; Wang X. et al., 2018). Interestingly, the miR-210 serum levels were additionally reported to decline after curative surgery was performed (Fedorko et al., 2015; Wang X. et al., 2018). The serum miR-210 levels could successfully differentiate RCC patients form healthy individuals; however, both sensitivity and specificity are strongly varying and are 81 and 79.4% (Zhao et al., 2013), 67.5 and 70% (Wang X. et al., 2018), and 65 and 83% (Iwamoto et al., 2014) in the different studies, respectively. However, cutoffs for biomarker dichotomization were determined using ROC curve analysis, which makes them difficult to compare. Moreover, no validation cohorts for diagnostic testing were implemented in the studies (Zhao et al., 2013; Iwamoto et al., 2014; Wang X. et al., 2018). Therefore, although there is strong evidence for a potential clinical utility of miR-210 serum levels, future studies need to address these issues. Interestingly enough, serum-derived exosomal miR-210 may offer additional insight for its biomarker potential in comparison to free circulating miR-210 (Wang X. et al., 2018; Zhang et al., 2018). As expected, miR-210 was again upregulated in RCC tissues in a study by Wang X. et al. (2018), which also matched the patient’s serum samples. This indicates that changes of miR-210 expressions in RCC cells are perfectly reflected by miR-210 circulating in the patient’s blood. However, there may be significant enrichment differences between exosomal and free circulating miR-210, as the latter is increased to a comparably lesser degree. This is also reflected in the diagnostic capability of the two approaches as indicated by significantly varying sensitivity and specificity for exosomal (AUC = 0.8779, 82.5, and 80%) and exosome-free (AUC = 0.7888, 67.5, and 70%) miR-210, respectively (Wang X. et al., 2018). Furthermore, the authors demonstrated increased exosomal miR-210 secretion of RCC cell lines under hypoxic experimental conditions (Wang X. et al., 2018), which is in line with another study investigating serum miR-210 levels in RCC (Dias et al., 2017). miR-210 is strongly connected to hypoxia as it is induced by the absence of functional von Hippel–Lindau (VHL) tumor suppressor gene, which is frequently encountered in RCC tumorigenesis (Shen and Kaelin, 2013), and by increasing hypoxia-inducible factor (HIF) activity (Neal et al., 2010; McCormick et al., 2013; Liu L. L. et al., 2017). Reciprocally, miR-210 may also target hypoxia-inducible factor 1α (HIF-1α), indicating a feedback loop, and thereby suppress hypoxia-induced apoptosis (Liu L. L. et al., 2017). miR-210 upregulation under hypoxic conditions results in increased proliferation, genetic instability, angiogenesis, and metastasis and adapts mitochondrial function (Dang and Myers, 2015). An increased release of miR-210 was observed when exposing RCC cell lines to hypoxic conditions in the previously discussed study by Dias et al. (2017), corroborating these results.

Fedorko et al. (2015) also demonstrated the diagnostic potential of serum miR-210; however, in their study, significantly elevated serum levels of miR-378 were better able to differentiate RCC patients from healthy controls with AUCs of 0.74 and 0.82 for miR-210 and miR-378, respectively. Nonetheless, the discriminatory ability of miR-210 and miR-378 combined performed best and discriminated RCC cases with a sensitivity and a specificity of 80 and 78%, respectively (Fedorko et al., 2015). Even so, evidence for the utility of miR-378 in blood-based liquid biopsy is not unambiguous. Although Hauser et al. (2012) could identify serum miR-378 to be significantly higher in a set of ccRCC patients and could discriminate cancer patients form healthy controls, they could not verify their results in the validation cohort. Furthermore, another study that aimed to evaluate a diagnostic panel including five miRNAs reported the miR-378 levels to be reduced in RCC patients as compared to healthy controls (Wang et al., 2015). As opposed to this, Redova et al. (2012) again reported that miR-378 serum levels significantly increased in patients with RCC, which they could further verify in a validation cohort. Apart from miR-378, Redova et al. (2012) also analyzed the diagnostic capability of miR-451 which was found to be significantly decreased in patient serum. This was also verified in a validation cohort. However, interestingly, the combination of miR-451 with the formerly discussed miR-378 could better differentiate the serum samples of healthy individuals from RCC patients with a sensitivity of 81% and a specificity of 83% (Redova et al., 2012). The varying and partly conflicting results regarding serum miR-378 may be due to differences in cohort size as well as baseline characteristics, yet collectively the clinical utility of serum miR-378 in RCC diagnostics at that time is uncertain. In RCC, the pathogenetic role of miR-378 has not been entirely resolved; however, miR-378 may be a regulator of angiogenesis, but this hypothesis is lacking a clear experimental *in vitro* and *in vivo* evidence (Li H. C. et al., 2014). In other cancer types, a pro-angiogenetic effect of miR-378 was demonstrated (Lee et al., 2007; Krist et al., 2015), and it could serve as a biomarker for antiangiogenetic therapy in ovarian cancer (Chan et al., 2014). Its role as an oncogenic or tumor-suppressive miRNA seems to vary depending on the cancer type (Zhang et al., 2014; Chen et al., 2016; Li S. et al., 2017; Zeng et al., 2017; Ma et al., 2019), which is frequently observed in other miRNAs (Svoronos et al., 2016).

As already mentioned before, Wang et al. (2015) developed a diagnostic miRNA signature enabling the diagnosis of early ccRCC, which, besides miR-378, also includes miR-193a-3p, miR-362, miR-572, and miR-28-5p. Apart from miR-378 and miR-28-5p, which were significantly decreased, the other miRNAs contained in the diagnostic panel were significantly overexpressed in the serum of ccRCC patients. The panel was able to differentiate patients with ccRCC from non-cancerous controls, especially at the early stages of the disease, accounting for 80% sensitivity and 71% specificity in stage I ccRCC (Wang et al., 2015). However, since corresponding tissue samples were not analyzed in the study, conclusions on the altered expression levels in RCC tissue and their potential reflection in serum miRNA expression and additional involvement in ccRCC pathogenesis cannot be drawn. As for miR-193a-3p, it was shown to target the tumor suppressor phosphatase and tensin homolog (PTEN) and mediate the PI3K/Akt pathway, thereby promoting RCC cell growth and progression (Liu L. et al., 2017; Pan Y. et al., 2018). miR-572 likewise represents an oncomir in RCC pathogenesis by enhancing proliferation and invasion and inhibiting tumor cell apoptosis by suppressing NF2/Hippo signaling (Guan et al., 2018; Pan et al., 2018a). On the other hand, miR-28-5p (Wang et al., 2016) and miR-362 (Zou et al., 2016; Zhu et al., 2020) were suggested as tumor-suppressive miRNAs in RCC, although the miR-362 serum levels were increased in the study by Wang et al. (2015).

Another miRNA that was reported to qualify as liquid diagnostic biomarker is miR-144-3p, which is overexpressed in the plasma of RCC patients and could successfully discriminate RCC from both healthy individuals and patients with renal angiomyolipoma. The sensitivity and the specificity were at 87.1 and 83.02% or 75 and 71.7%, respectively (Lou et al., 2017). The miR-144-3p levels in RCC tissue were likewise increased, and the plasma levels drastically dropped after curative surgery was performed (Xiao et al., 2017). miR-144-3p may promote ccRCC progression and induce resistance to the receptor tyrosine kinase inhibitor sunitinib by targeting AT-rich interaction domain 1A (ARID1A), which is the gene of the SWI/SNF subunit in the SWI/SNF chromatin remodeling complex (Xiao et al., 2017; Mathur, 2018). ARID1A influences cell cycle, apoptosis, and p53 targets, thus acting as a tumor suppressor (Mathur, 2018). However, the role of miR-144-3p in RCC pathogenesis may not be clear as another study conversely suggested miR-144-3p both being decreased in RCC tissue and acting as a tumor suppressor by targeting mitogen-activated protein kinase 8 (MAP3K8) (Liu F. et al., 2016).

miR-508-3p and miR-509-5p were both demonstrated to play a role in RCC pathogenesis and additionally altered tissue and plasma expression levels were reported. As for miR-508-3p, the expression levels in tissue, plasma, and serum are consistently decreased as reported by two studies (Zhai et al., 2012; Liu et al., 2019a), and serum miR-508-3p was able to differentiate RCC and non-cancer samples (AUC = 0.8), which even improved when combined with increased serum levels of miR-885-5p (AUC = 0.9) (Liu et al., 2019a). As for miR-509-5p, the evidence is not as clear since its expression was found to be decreased in the plasma of RCC patients, yet the difference was not significant in every available study although the baseline characteristics of the study population were similar (Zhai et al., 2012; Zhang et al., 2013). Nonetheless, both miR-508-3p and miR-509-5p may act as tumor suppressors in RCC by inducing apoptosis and inhibiting cell proliferation and migration *in vitro* (Zhai et al., 2012; Zhang et al., 2013; Liu et al., 2019a). Zinc finger E-box-binding homeobox 1 and nuclear factor kappa B subunit 1 may represent targets of miR-508-3p as demonstrated in cervical and gastric cancer (Huang et al., 2016; Guo et al., 2018).

Furthermore, Chen et al. (2018a) also investigated several plasmatic miRNAs for their diagnostic potential in RCC, including miR-210, which we already discussed earlier. In their study, besides miR-210, the authors included miR-224 and miR-141 and analyzed various plasmatic combinations of the three miRNAs. Interestingly, the combination of the significantly elevated miR-224 and the decreased miR-141 plasma levels in a miR-224/miR-141 ratio showed the best diagnostic capability (AUC = 0.9898, sensitivity 97.06%, specificity 98.53%), representing a promising potential diagnostic tool in RCC patients (Chen et al., 2018a). However, this was not tested in a validation cohort, and external validation is missing. Considering the varying and partly conflicting results in the analysis of miRNAs in a larger body of evidence, at this point, it should be considered hypothesis-generating until confirmed by future studies.

miR-21, which has been repeatedly shown to be overexpressed in RCC tissue and has been suggested as a prognostic marker in RCC patients (Faragalla et al., 2012; Zaman et al., 2012; Vergho et al., 2014), was also reported to be elevated in patients’ serum samples and positively correlated to tumor stages, indicating a potential role as a blood-based diagnostic biomarker (Cheng et al., 2013; Tusong et al., 2017). Additionally, a decline in miR-21 serum levels after surgery was reported (Tusong et al., 2017). However, Sanders et al. (2012) could not confirm the potential clinical value of miR-21, as, in their analysis, the expression levels were highly similar between controls and urological cancers including RCC. This may be due to differences in the study populations as serum miR-21 could be correlated to tumor stages (Cheng et al., 2013). Interestingly, increased serum levels of miR-21 were observed in other cancer types as well, including prostate, lung, and breast cancer (Zhang H. L. et al., 2011; Liu et al., 2012; Schwarzenbach et al., 2012). Besides miR-21, Cheng et al. (2013) also discovered that miR-34a and miR-224 were overexpressed, and miR-141 expression was significantly decreased in the sera of RCC patients, corroborating previously discussed results of miR-224 and miR-141 (Chen et al., 2018a).

In RCC cells, miR-21 was proposed to act as an oncogenic miRNA and enhance tumor progression and proliferation while inhibiting apoptosis (Zhang A. et al., 2011; Li X. et al., 2014; Petrozza et al., 2015). Additionally, it may target VHL mRNA (Sun et al., 2019; Zang et al., 2019), the loss of which plays a major role in RCC tumorigenesis and promotes normoxic HIF signaling (Shen and Kaelin, 2013).

miR-106a is another candidate for a diagnostic miRNA in ccRCC as it was significantly overexpressed in the serum of 30 ccRCC patients with matched controls and was reduced after curative surgery, which indicates a potential use in post-surgery biomarker monitoring (Tusong et al., 2017). In addition, miR-106a could differentiate ccRCC patients from controls with an AUC of 0.819 and a sensitivity and a specificity of 86.7 and 70%, respectively (Tusong et al., 2017). However, the members of the miR-106 family miR-106a-5p and miR-106a^∗^ were previously reported to be downregulated in RCC tissue (Ma et al., 2015; Pan et al., 2017), which is in contrast to the findings of increased miR-106a levels in patient serum by Tusong et al. (2017). miR-106a-5p overexpression was shown to inhibit both cell migration and invasion *in vitro* and reduced metastases *in vivo* by targeting p21 activated kinase 5 (Pan et al., 2017). Moreover, miR-106a^∗^ was found to suppress the proliferation of RCC cell lines by inhibiting IRS-2 and thus inhibiting PI3K/Akt signaling (Ma et al., 2015). Therefore, the serum expression of miR-106a should be interpreted with caution unless validation in larger cohorts was performed.

Another miRNA which may qualify as a future marker in the diagnosis of RCC is miR-625-3p. It was reported to be overexpressed in ccRCC tissues yet decreased in the serum of ccRCC patients (Zhao et al., 2019a). A high tissue expression of miR-625-3p was an independent predictor for poor OS (HR = 2.949, 95%CI 1.413–6.152, *p* = 0.004), whereas serum expression could significantly differentiate ccRCC patients from healthy controls (AUC = 0.792, sensitivity 70.3%, specificity 80%) (Zhao et al., 2019a). However, the inconsistency in miR-625-3p expression in tumor tissue and patient serum demands further validation. In functional investigations in RCC cell lines, miR-625-3p was shown to enhance RCC cell migration and invasion and additionally inhibit apoptosis (Zhao et al., 2019a).

Wulfken et al. (2011) identified miR-1233, which showed significantly higher levels in both RCC tissue and patients’ serum, as a biomarker candidate in RCC diagnostics. The diagnostic utility was verified in a multicenter validation cohort including 84 RCC patients and 93 controls, yet although miR-1233 was able to significantly discriminate RCC patients from healthy individuals, the results in the validation cohort may not support its use in clinical routine (AUC = 0.588, sensitivity 77.4%, specificity 37.6%) (Wulfken et al., 2011). Even so, the combination of miR-1233 with miR-141 could promisingly enhance the diagnostic capability, reaching a sensitivity of 100% and a specificity of 73.3% (Yadav et al., 2017). In addition, Zhang et al. (2018) found that serum exosomal miR-1233 increased in their study including 82 RCC patients and successfully differentiated RCC from non-cancer samples, corroborating previous results. The pathogenetic role of miR-1233 in RCC to date is not well investigated; however, the observed increase of miR-1233 levels upon hypoxia in RCC cell lines indicates its involvement in hypoxic cell signaling (Dias et al., 2017).

Apart from acting as biomarkers, exosomal miRNAs were suggested to promote RCC progression. Wang et al. (2019) reported that exosomal miR-19b-3p that originated from ccRCC cancer stem cells (CSC) in RCC metastases transfers EMT potential to renal cancer cells by being incorporated by the tumor cells. Mechanistically, miR-19b-3p targets the tumor suppressor PTEN and enhances lung metastases (Wang et al., 2019). This was also verified in rodent models, and CD103 overexpression in CSC exosomes was suggested to guide exosomes to their destinations in tumor and lung tissue. Corroborating these results, in a cohort of 209 ccRCC patients, CD103+ exosomes were significantly increased in patients with metastatic disease, indicating a potential use in the diagnosis and the monitoring of metastasized ccRCC (Wang et al., 2019).

### Circulating miRNAs as Prognostic Biomarkers in RCC

Dias et al. (2017) aimed to analyze the potential prognostic utility of the already afore-discussed miR-210, miR-221, and miR-1233 in the blood plasma of RCC patients. Interestingly, the increased plasma levels of all investigated miRNAs were significantly associated with poor cancer-specific survival (CSS) and independently predicted for CSS when combined as a single biomarker (HR = 3.02, 95%CI 1.19–7.64, *p* = 0.014). Moreover, this miRNA panel complements prognostic parameters, such as tumor stage, Fuhrman grade, age, and gender, as it significantly improves their prognostic capacity when combined to an index with already established clinico-pathological parameters (Dias et al., 2017). As for miR-221, this corroborates the findings of Teixeira et al. (2014) who reported miR-221 to be upregulated in the plasma of RCC patients. Moreover, plasma levels were found to be even higher in patients who already had metastatic disease when first diagnosed with RCC. Increased plasmatic miR-221 expression levels represented an independent prognostic marker for OS (Teixeira et al., 2014). In the same study, miR-222, the plasmatic expression of which was also observed to be increased in RCC patients *versus* healthy controls, did not validate as an independent predictor of overall survival (OS). Interestingly, the addition of plasmatic miR-221 levels to the already established clinico-pathological parameters TNM stage, Fuhrman grade, and age combined could improve their prognostic capability (c-index 0.8, HR = 4.5, 95%CI 1.17–17.34, *p* = 0.029 *versus* c-index 0.961, HR = 10.7, 95%CI 1.33–85.65, *p* = 0.26) (Teixeira et al., 2014). Both miR-221 and miR-222 were found to be significantly overexpressed in RCC tissue *vs.* healthy renal parenchyma and have already been considered as promoters of RCC proliferation and progression in numerous studies (Lu et al., 2015; Liu et al., 2019b; Zhao et al., 2019b; Lv et al., 2020). Furthermore, both miRNAs were proposed as predictive markers for treatment response to sunitinib therapy and were demonstrated to enhance angiogenesis *via* targeting the vascular endothelial growth factor receptor 2 (VEGFR2) (Khella et al., 2015; Garcia-Donas et al., 2016). This mechanism was also shown to regulate the response to sunitinib treatment in prostate cancer (Krebs et al., 2020). miR-221 was additionally suggested in the regulation of HIF-1α, which plays a major role in RCC pathogenesis (Penolazzi et al., 2019), and beyond that, it could enhance RCC cell proliferation, migration, and invasion by targeting the tissue inhibitor of metalloproteinases 2 (TIMP2) as found in cell culture models (Lu et al., 2015).

miR-224 was already shown to have a potential diagnostic utility in the previous chapter (Chen et al., 2018a). As for its impact on RCC prognosis, Fujii et al. (2017) analyzed exosomal miR-224 in RCC patients. miR-224 was upregulated in RCC tissue and the high exosomal miR-224 in the blood of 108 RCC patients independently predicted for shorter CSS, progression-free survival (PFS), and OS (CSS: HR = 1.6, 95%CI 1.1–2.5, *p* = 0.014; PFS: HR = 11, 95%CI 3.3–68.7, *p* ≤ 0.0001; OS: HR = 9.1, 95%CI 1.8–166.1, *p* = 0.0043), respectively (Fujii et al., 2017). Functional experiments revealed an oncogenic role of miR-224 in RCC pathogenesis. Exosomes containing miR-224 from a metastatic RCC cell line that were added to a cell line of primary RCC enhanced proliferation and invasion and, moreover, increased the intracellular miR-224 levels in primary cell lines (Fujii et al., 2017). This indicates the importance of exosomal miRNAs in cancer progression (Fujii et al., 2017; Kulkarni et al., 2019). Furthermore, miR-224 was shown to influence RCC progression by directly targeting alpha-2,3-sialyltransferase IV and impacting the PI3K/AKT pathway (Pan Y. et al., 2018).

Another study that investigated the prognostic utility of plasma exosomal miRNAs was conducted by Du et al. (2017). In their study, in a first step, plasma exosomal miRNA expression levels were evaluated in a discovery cohort including 44 patients with metastatic RCC. In a second step, the most promising miRNAs were confirmed in a validation cohort of 65 patients. Eventually, miR-26a-1-3p (HR = 0.43, 95%CI 0.1–0.84, *p* = 0.025), miR-615-3p (HR = 0.36, 95%CI 0.11–0.54, *p* = 0.0007), and miRNA let-7i-5p (HR = 0.49, 95%CI 0.21–0.84, *p* = 0.018) retained their prognostic value in the validation set, and decreased plasma exosomal expression was significantly associated with adverse prognosis (Du et al., 2017). Moreover, Du et al. (2017) compared the prognostic ability of the established Memorial Sloan-Kettering Cancer Center (MSKCC) score, which takes multiple clinico-pathological factors into consideration, *versus* the MSKCC score with the inclusion of let-7i-5p. In their analysis, the addition of let-7i-5p improved the prognostic capability (AUC = 0.64) as compared to the MSKCC score alone (AUC = 0.58) (HR = 3.43, 95%CI 2.73–24.15, *p* = 0.0002 for the combination) (Du et al., 2017). The miRNA let-7 family mainly exerts tumor-suppressive functions in cancer, yet several subtypes may also act as oncogenes (Chirshev et al., 2019). However, let7i-5p and its role in RCC pathogenesis have not been evaluated yet. The members of the let7-family let7b and let7c were reported to be decreased in RCC tissue and were able to suppress proliferation and restore chemoresistance to 5-fluorouracile in *in vitro* experiments (Peng et al., 2015). In contrast, let-7g-5p and let-7i-5p were significantly overexpressed in a study including 94 ccRCC specimens (Gowrishankar et al., 2014). The differing results across the let-7 subtype underline the different biological roles of let-7 subtypes, and further research is therefore necessary to clarify the functional role of exosomal let-7i-3p in RCC. Results in other cancer types suggest a tumor-suppressive role of let-7i as, for instance, they are downregulated in ovarian and bladder cancer and inhibited tumor proliferation and chemoresistance (Yang et al., 2008; Qin et al., 2019). As for miR-615-3p, Wang Q. et al. (2018) found it to be sponged by the lncRNA HOXA transcript at the distal tip (HOTTIP) and to target insulin-like growth factor 2 in RCC. In their study, HOTTIP was shown to be upregulated in RCC tissue, and the overexpression of HOTTIP was reported to promote RCC proliferation, migration, and invasion *in vitro*, implicating a tumor-suppressive role of miR-615-3p (Wang Q. et al., 2018).

Chanudet et al. (2017) screened for circulating miRNAs in RCC using microRNA arrays in plasma samples of 94 ccRCC patients of all clinical stages and 100 healthy controls. Although they could not recommend single miRNAs or miRNA signatures for the early diagnosis of ccRCC, thereby contradicting previous results, the authors found lower miR-150 expression in the plasma of ccRCC patients to be independently associated with cancer-specific survival (HR = 1.3, 95%CI 1.0–1.8, *p* = 0.03) (Chanudet et al., 2017). Even though miR-150 has not been functionally investigated in RCC yet, the utility of exosomal serum miR-150 levels in colorectal cancer for prognosis prediction supports the notion of its future clinical utility in RCC (Zhao Y. J. et al., 2019; Zou et al., 2019). However, miR-150 may exert different functions in different cancer types as, for instance, it promotes metastasis in lung cancer, whereas it may behave as a tumor suppressor in colorectal cancer (Li et al., 2016; Chen et al., 2018b).

### Circulating miRNAs as Predictive Biomarkers for Therapy Response

Circulating miRNAs might also be involved in acquiring resistance to treatment regimens in RCC. A recent study suggests that miR-35-5p carried in extracellular vesicles participates in the development of resistance to the multi-targeted receptor tyrosine kinase inhibitor sunitinib (He et al., 2020). In the study, sorafenib-sensitive cell lines were exposed to extracellular vesicles of sorafenib-resistant RCC cells, which contained increased levels of miR-31-5p. This resulted in increasing sorafenib resistance in the former treatment-sensitive cell lines, indicating transmitted therapy resistance by miR-31-5p *via* extracellular vesicles. The role of miR-31-5p in the induction of sorafenib resistance was subsequently demonstrated in further *in vitro* and *in vivo* experiments, and functional analysis revealed MutL homolog 1 (MLH1) as a direct target of miR-31-5p. The authors speculate that reduced MLH1 expression and subsequent increased genomic instability may facilitate the development of drug resistance (He et al., 2020). Ultimately, He et al. (2020) found significantly increased levels of miR-31-5p within extracellular vesicles in the serum of RCC patients, who had progressed on sorafenib-based treatment, as compared to serum levels before treatment initiation. This indicates a possible role as a predictive biomarker for the development of sorafenib resistance; however, the authors did not further investigate miR-31-5p levels and the potential impact on clinical endpoints such as PFS or response rates (He et al., 2020). Nevertheless, the role of miR-31-5p in RCC pathogenesis is not without inconsistencies. A previous study suggested that miR-31-5p may act as a tumor suppressor in RCC by inhibiting proliferation, migration, and invasion *via* targeting cyclin-dependent kinase 1 (Li et al., 2019). Moreover, the expression of miR-31-5p has been reported to be significantly lower in RCC tissue and cell lines (Li et al., 2019).

Gamez-Pozo et al. (2012) developed several predictive models of miRNA expression in the peripheral blood for resistance to sunitinib therapy in a prospective observational multicenter study including 38 metastatic RCC patients who received first-line treatment with sunitinib. Blood samples were taken before and 2 weeks after treatment initiation, and micro-array assays and subsequent verification of promising miRNA candidates with qPCR were performed. The authors developed 12 predictive models for each response group, whereas poor response was defined as progression earlier than 6 months and prolonged response was defined as progression later than 18 months after therapy initiation. The expression levels of up to four miRNAs per individual model and 28 miRNAs in total were significantly related with their respective prognostic groups. One model of the poor response group, including miR-192, miR-193-3p, and miR-501-3p, and one model of the prolonged response group, which included miR-miR-410, miR-1181, and miR-424^∗^, prevailed their predictive ability in the validation with qRT-PCR (Gamez-Pozo et al., 2012). However, due to the relatively small patient sample, further research and validation is required.

## Circulating Long Non-Coding RNAs in RCC

Although the utility of circulating lncRNAs as biomarkers in cancer and potential pathomechanisms have been reported repeatedly, in RCC data remain limited.

The lncRNA gradually increased during hepatocarcinogenesis (GIHCG) may represent a promising biomarker in RCC (He et al., 2018). GIHCG is overexpressed in RCC tissue as compared to healthy adjacent tissue and is also elevated in the serum of RCC patients, showing a highly significant correlation with tumor tissue expression levels. Moreover, the GIHCG serum levels were able to discriminate RCC patients and healthy individuals with a sensitivity and a specificity of 87 and 84.8%, respectively (AUC = 0.920) (He et al., 2018). In addition, GIHCG retained its diagnostic ability when only early-stage RCC and healthy controls were compared, and RCC tissue expression levels were significantly associated with OS; however, no uni- and multivariate analyses were performed (He et al., 2018). GIHCG was first described in hepatocellular carcinoma, where it was demonstrated to enhance tumor cell proliferation, migration, and metastases both *in vitro* and *in vivo* by recruiting the transcription factors EZH2 and DNMT1 to the miR-200b/a/429 promoter, thereby epigenetically silencing miR-200b/a/429 expression (Sui et al., 2016). Since then, GIHCG’s oncogenic role has been reported in various cancers such as ovarian, colorectal, gastric, and cervical cancer (Yao et al., 2018; Jiang X. et al., 2019; Liu G. et al., 2019; Zhang et al., 2019). He et al. (2018) eventually confirmed this role in RCC in knockdown experiments.

Wu et al. (2016) attempted to develop a panel including several lncRNAs as a novel diagnostic marker for RCC. In their study, potential lncRNAs for further investigation were first discovered in RCC tissue, and the serum expression of lncRNA candidates was analyzed in a discovery cohort including 25 patients with ccRCC, pRCC, or chRCC in a second step. Subsequently, promising lncRNAs were evaluated in a validation set of 37 patients and 35 healthy controls. A panel of five lncRNAs, low expressed in tumor (LET), plasmacytoma variant translocation 1 (PVT1), promoter of CDKN1A antisense DNA damage activated RNA (PANDAR), phosphatase and tensin homolog pseudogene 1 (PTENP1), and linc00963, was able to successfully discriminate non-cancer from RCC patients (AUC = 0.823) and retained their diagnostic ability throughout the different clinical TNM stages, also indicating their utility as early markers for RCC (Wu et al., 2016). In an additional small clinical patient set which also included patients with benign renal tumors, the cancer patients showed significantly higher risk indices as compared to controls (Wu et al., 2016). lncRNA LET is considered a tumor suppressor in a variety of cancer types which also accounts for RCC, where LET was shown to enhance apoptosis and impair mitochondrial membrane potential by targeting miR-373-3p, thereby regulating Dickkopf 1 and tissue TIMP2 expression (Weidle et al., 2017; Ye et al., 2019). Moreover, PTENP1, too, acts as a tumor-suppressive factor in RCC by acting as a competing endogenous RNA for miR-21, which would otherwise promote cell proliferation, migration, and invasion by the suppression of PTEN, an important tumor suppressor (Zhang A. et al., 2011; Song et al., 2012; Yu et al., 2014). In contrast, PVT1 (Pan et al., 2018b; Derderian et al., 2019) and PANDAR (Li J. et al., 2017; Han et al., 2019) are well-known oncogenic lncRNAs, and their role in RCC tumorigenesis and negative impact on RCC outcome have been reported (Bao et al., 2017; Xu et al., 2017; Yang et al., 2017; Li et al., 2018). Linc00963 has not been investigated in RCC yet but was indicated to promote cancer progression in a variety of malignancies such as head and neck cancer osteosarcoma and breast cancer (Zhou et al., 2019b; Lee et al., 2020; Wu et al., 2020). However, Wu et al. (2016) found that the expression levels of PVT1 and linc00963 were significantly downregulated in the serum of RCC patients as compared to tissue expression levels.

The lncRNA activated in RCC with sunitinib resistance (ARSR) was first described by Qu et al. (2016), who suggested the involvement of exosomal ARSR in the development of resistance to sunitinib in RCC and discovered its potential pathomechanism. ARSR expression was increased in the RCC tissue of patients who were resistant to sunitinib as well as in resistant RCC cell lines. Moreover, plasma levels were significantly elevated in RCC patients as compared to healthy controls, and detectable ARSR levels dropped after surgical treatment and increased upon tumor recurrence. Higher plasma ARSR levels independently predicted for shorter PFS for sunitinib treatment (HR = 2.9, 95%CI 1.2–7.1, *p* = 0.017) (Qu et al., 2016). This indicates that the circulating ARSR in RCC patients is a promising biomarker for response to sunitinib (Qu et al., 2016). Interestingly, ARSR has also been associated with drug resistance in osteosarcoma and hepatocellular carcinoma (Li Y. et al., 2017; Shen and Cheng, 2020). Furthermore, Qu et al. (2016) elucidated the underlying mechanism in RCC and demonstrated that ARSR upregulation is indispensable for the development of sunitinib resistance. ARSR was shown to sponge both miR-34 and miR-449, thereby preventing them from binding to their targets AXL and c-MET, thus inhibiting their degradation. As a result, ARSR upregulation results in a likewise increase of AXL and c-MET and further activation of the downstream STAT3, AKT, and ERK signaling pathway (Qu et al., 2016). The involvement of ARSR in STAT3 and AKT regulation was confirmed in liver cancer (Li Y. et al., 2017; Yang et al., 2019). Moreover, in RCC, AKT was found to promote ARSR expression by inhibiting forkhead box protein O1/3a (FOXO1 and FOXO3a), which results in the formation of a positive feedback loop (Qu et al., 2016). Interestingly, the exosomal secretion of ARSR was reported to transfer sunitinib resistance to former sunitinib-sensitive cells both *in vitro* and *in vivo*, indicating the exosomal dissemination of sunitinib resistance. In addition, therapeutically targeting ARSR could restore sunitinib sensitivity *in vivo* (Qu et al., 2016).

## Circulating YRNAs in RCC

YRNAs are small ncRNA molecules that form characteristic stem-loop formations because of their complementary 5′ and 3′ UTRs (Pruijn et al., 1993). Originally, they were reported in rheumatic diseases where they were found to be part of soluble ribonucleoproteins (Hendrick et al., 1981). YRNAs comprise the four subtypes hY1, hY3, hY4, and hY5 and are abundant in both serum and plasma (Dhahbi et al., 2013; Yeri et al., 2017). Recently, they have been suggested as circulating biomarkers in breast as well as head and neck cancer (Dhahbi et al., 2014; Victoria Martinez et al., 2015). In RCC, although Nientiedt et al. (2018) reported significant alterations in the expression levels of hY3 and hY4 in malignant *versus* healthy tissue, they could not find any differences in serum expression levels. Thus, YRNAs do not bring any additional value as easily available blood-based biomarkers in RCC (Nientiedt et al., 2018).

## Comparison to Current Standards

The early diagnosis of RCC still remains difficult due to the lack of broad screening programs and a specific panel of symptoms already noticeable at early-stage disease. Many patients are diagnosed incidentally when they present with symptoms like hematuria, abdominal mass, or flank pain (Vasudev et al., 2020). Since many of these symptoms are non-specific, the majority of patients do not get completely evaluated within the first 6 months after initial presentation (Zhou et al., 2019a), thus reducing the number of patients diagnosed at an early stage. This is a major issue since early diagnosis is crucial for successful therapy and is associated with better patient survival (Tsui et al., 2000; Palsdottir et al., 2012). Hence, finding liquid biomarkers for early-stage disease detection might prove to be beneficial for patients. Due to the minimal invasiveness of liquid biopsies, they could be performed routinely. However, up to now, there is no established biomarker available for clinical use. RCC is reported to present with lower circulating tumor DNA (ctDNA) levels compared to other tumor entities (Smith et al., 2020), excluding it as a feasible diagnostic marker in RCC. Also, CTCs have not been established for clinical use in RCC diagnosis yet due to heterogenous surface marker expression (Bu et al., 2020), which makes an enrichment with the CellSearch^TM^ system, the only FDA-approved CTC capture platform, difficult (Bai et al., 2018). Therefore, circulating ncRNAs might represent useful and easily available biomarkers in the diagnosis and treatment of RCC. As summarized in this review, the analysis of various circulating ncRNAs can distinguish RCC patients from healthy donors. However, systematic screenings and larger-scale studies, which would be necessary for a potential clinical implementation in the diagnosis of early-stage RCC, are missing.

For prognostic purposes, the International Metastatic RCC Database Consortium (IMDC) and MSKCC risk scores are the current standard tools in RCC. Both use a panel of six or five clinical parameters to predict patient outcome, respectively (Tanaka et al., 2016). Even though they are widely validated, there are also reports showing their limitations. Indeed they performed fairly accurate in predicting OS in different cohorts of mRCC patients undergoing first- and second-line targeted therapy (Heng et al., 2009; Ko et al., 2015; Tanaka et al., 2016). Their respective prognoses for around 23% of patients in a treatment-naïve cohort undergoing the first line of targeted therapy do not match, suggesting limited prognostic capacity (Okita et al., 2019). They also do not perform well in predicting OS in patients undergoing cytoreductive nephrectomy (Westerman et al., 2020). Thus, implementation of additional or new biomarkers, to reliably stratify RCC patients in the era of targeted therapies, might be beneficial. It was already shown that the inclusion of an additional miRNA as a marker could improve the prognostic capacity of the MSKCC score (Du et al., 2017). Various ncRNAs, as described in this review, have prognostic capacities for OS (Teixeira et al., 2014) or therapy response (Gamez-Pozo et al., 2012; Li et al., 2019; He et al., 2020). In general, multiple miRNAs or lncRNAs summarized in a distinctive panel or signature appear to bring higher predictive values. Hence, comparisons of ncRNAs alone or in combination with well-established clinical scores, with scores like the MSKCC and IMDC, might be interesting to evaluate to check whether prognosis prediction in various settings can be improved.

## Conclusion

In order to make circulating ncRNAs feasible biomarkers for diagnosis and prognosis in RCC, comparisons with current, well-established standards are necessary to determine whether they add any value. Therefore, sensitivity, specificity, and accuracy would have to be compared or added to scores like the IMDC or the MSKCC or other promising methods such as genomic profiling.

However, due to missing validations and an ongoing discussion about, for example, optimal sample processing and normalization, circulating ncRNAs are not yet to enter clinical practice in RCC, and further research is needed.

## Author Contributions

DB and MP conceptualized this work. DB contributed to visualization and writing—preparing the original draft. DB, RD, LO, LF, OS, GC, and MP contributed to writing—reviewing and editing the manuscript. MP supervised the study. OS, GC, and MP took charge of funding acquisition. All the authors have read and agreed to the published version of the manuscript.

## Conflict of Interest

The authors declare that the research was conducted in the absence of any commercial or financial relationships that could be construed as a potential conflict of interest.
